# Nurses’ comfort and well-being: A descriptive study to find out the relationship between nurses’ awareness of self-comfort and well-being at long-term care settings in the state of Kuwait

**DOI:** 10.1097/MD.0000000000037479

**Published:** 2024-03-22

**Authors:** Sally Mohammed Farghaly Abdelaliem, Hasna’a Salem Saed Boswihi

**Affiliations:** a Nursing Management and Education Department, College of Nursing, Princess Nourah bint Abdulrahman University, Riyadh, Saudi Arabia; b Faculty of Nursing, Nursing Administration Department, Alexandria University, Alexandria, Egypt; c Medical Rehabilitation Center, Public Authority for the Disabled Affairs, Kuwait.

**Keywords:** comfort, Kuwait, long-term care, nurses, relationship, well-being

## Abstract

The study aims to examine out nurses’ awareness of self-comfort and well-being in long-term care settings in the state of Kuwait to avoid profession burnout. Nurses are vital resource to health care institutions, the attention on nurses’ comfort and well-being should be given valued concerns by top management, as poor staff well-being can pose risks among them and may result in poor performance such as less quality care that is result from less productivity and if the staff well-being ignored the institution performance might be underachieved. A descriptive cross-sectional study design was utilized. The questionnaire assessed participants’ socio-demographic data, comfort, and well-being. Data were collected from 260 (86.7% response rate) nurses over the course of 3 months from long-term care facilities in 2 different organizations in Kuwait. The overall nurses’ comfort level was moderate with a mean percent score of 67.55 ± 12.50. However, the overall nurses’ well-being level was low with a mean percent score of 49.58 ± 6.93. As well as, a strong, positive, and significant correlation was noticed between overall nurses’ comfort and its domains with the overall nurses’ well-being and its domain. The regression analysis revealed that nurses’ perception of their comfort may predict their well-being (*P* < .001) and explained 34% of the variation in nurses’ well-being (*R*^2^ = 0.470, *β* = 0.428, *F* = 34.762, *P* < .001). This study emphasized on the importance of creating a healthy, comfortable work environment that promotes nurses’ well-being, as well as eliminating discomfort and job dissatisfaction from the organizational culture among nurses as it effects on the organizational performance, productivity, and quality of care.

## 1. Introduction

When nurses are under physical or emotional stress and do not report what is wrong, their performance suffers, which has an impact on patient care and the performance of the healthcare organization. On the contrary, when their comfort is increased, they are happier with their job, their performance is better, absenteeism and turnover are reduced, and as a consequence, the health care institution is strengthened and patients receive superior treatment.^[1]^ Healthcare organizations vary in size and type of care delivered, and nurses face a variety of stressful scenarios such as working long hours, an exhausting work schedule, unpleasant working conditions, understaffing, patient mortality, and dealing with contagious infections. Recently, while the media has focused on the services of physicians and nurses working in Kuwait Ministry of Health hospitals, long-term care (LTC) nurses have mostly gone unnoticed.^[2]^

LTC nurses face the same stresses as nurses working in hospitals, with the exception that they interact with residents who have chronic physical and/or mental disorders, who are mostly elderly or have developmental disabilities.^[3]^ Those residents require significant care since they are unable to live independently; they require assistance with daily living tasks for the majority of the day. LTC nurses get to know the patients and their families and establish significant relationships as they offer care at all hours of the day and night for months, if not years. As a result, they are emotionally linked to the residents and do their best to provide excellent care.^[3]^

LTC nurses are frequently stressed due to a low staff level, as there may be only 1 nurse on duty at times, resulting in physical and mental exhaustion.^[3]^ They understand that providing care entails not only assisting residents with daily activities, but also attending to their social and emotional needs. These facets are vital to quality care because they are essential to recognizing the humanity of the residents, the sort of care offered for people in LTC leaves them with no time to relax, resulting in mental and physical exhaustion.^[4]^

LTC nurses in Kuwait face the same stress that LTC nurses in other nations do, they typically work with people who are physically or mentally challenged, as well as those who are uncooperative. Nurses face dissatisfying working conditions due to workflow, excessive working hours, and an unhealthy work environment. Furthermore, the large number of residents with cognitive disabilities who exhibit hostile conduct against staff or other residents is a major source of occupational stress for the staff.^[4]^

A new factor has compromised nurses’ physical and emotional well-being all across the world in 2020. It is the novel coronavirus pandemic infection (COVID-19). Nurses are shown to be the most nervous and stressed among healthcare employees while caring for and treating COVID-19 infected patients.^[5]^ The novel coronavirus ravaged the globe, infecting numerous countries, and temporarily paralyzing the whole planet. Nurses were among the frontline group to provide direct care for those infected with this virus, they were working 24/7 to provide care and comfort to the patients while also trying to avoid becoming infected, as a result, many nurses became exhausted and burned out due to the increased number of infected cases, death, lack of personal protective equipment and sanitizers, they are worried every day about whether they’ve contracted the disease or passed it on to their families and friends^.[6,7]^

The novel coronavirus was and continues to be a nightmare for healthcare workers, particularly nurses who provide direct care. The pandemic increased nurses’ stress and dread, resulting to increased turnover in most healthcare facilities, generating shortages all around the world. According to the International Council of Nurses, healthcare professionals account for around 10% of all cases worldwide, with over 20,000 afflicted. It was also stated that the outbreak claimed the lives of at least 1500 nurses and many other healthcare professionals, and that the epidemic has produced major physical and psychological issues for nurses who deal directly with sick or confined patients.^[8]^ A similar issue was documented during the SARS virus, the H1N1 pandemic flu, and the Ebola outbreak, generating psychological anguish in nurses and exacerbating the lack of nurses owing to resignation, sick leave, and mental illness.^[9]^

More importantly, poor mental health affects not just the health of nurses, but also their performance and the quality of patient care.^[10]^ At a time when COVID-19 infection is varying internationally due to viral mutations, nursing has become a physically and emotionally taxing occupation. Nurses in various healthcare settings are troops battling an active adversary to assist patients and residents in recovering from illness and protecting others, themselves, and their families from becoming infected; as a result, they encountered difficulties in carrying out their duties.^[11]^ The additional strain and physical hardship of wearing personal protective equipment were endangering nurses’ health. Nurses labored above their capacities and continue to do so due to a shortage of staff availability, which forced them to work long hours and accept a greater number of patients. Nurses who work long hours and are constantly stressed experience physical symptoms such as headaches, high blood pressure, and fatigue, as well as psychological symptoms such as depression, frustration, and mental dysfunction, which result in impaired performance and judgment, a negative attitude toward work, and indecisiveness.^[12,13]^ There is compelling evidence that high levels of occupational stress in general, and nursing stress in particular, are substantially linked to low levels of self-reported health and well-being.^[14]^

Nurses are vital resource to health care institutions, the attention on nurses’ comfort and well-being should be given valued concerns by top management, as poor staff well-being can pose risks among them and may result in poor performance such as less quality care that is result from less productivity and if the staff well-being ignored the institution performance might be underachieved.^[15,16]^ The comfort and well-being of nurses is critical to the success of the patients’/residents’ care and the performance of the healthcare facility. According to Nowell,^[17]^ when nurses’ comfort is improved, they are more contented, dedicated to the institution, and able to perform more efficiently. The outcomes of these nurses boosted organizational strength, as a result, nurses should understand that their comfort is essential for their well-being as well as for positive patient outcomes; they should be aware that working while they are uncomfortable or in distress will never provide quality care, and they will never be able to continue the process of care.^[18]^

The concept of “comfort” comes from the Latin word “confortare,” which means “to strengthen” therefore, the notion of comfort leads to an activity that results in comfort.^[19]^ It is a critical factor that has been defined in several nursing theories as follows: Orlando described comfort as “a reaction to human needs,” Leininger and Watson defended comfort as “important to the care process,” Peplau identified comfort as “a fundamental human need related with food, rest, and communication,” and Roy used comfort techniques to attain psychological comfort. According to Pinto et al,^[20]^ comfort is a “state of well-being that can occur at any point of the illness-health continuum.” Morse also depicted comfort as a momentary (e.g., pain management) or long-term (e.g., attainment of ideal health) condition, depending on the discomfort or need addressed. Metta^[21]^ defines comfort as personalized behavior that promotes health and healing. Nurses must be conscious of their lifestyle choices; health behaviors should be addressed in order to sustain daily functions and improve their health.

Health seeking behavior (HSB) is defined as “an individual act to promote maximum well-being, recovery, and rehabilitation, which could occur with or without health concerns and within a range of potential to real health concerns.” It is also defined as “an action in which an individual makes a decision to find a support to complete his/her personal need that is related to illness condition.” All behaviors connected with building and maintaining a healthy physical and mental state (primary prevention), dealing with any deviation from the healthy condition (secondary prevention), and lowering the effect and course of a disease are included in HSB (tertiary prevention). HSB can also be suggested by routine medical exams, healthcare facility selection, admittance when having health concerns, and consumption of health services. HSB enhanced by comfort that result in well-being.^[22]^

When nurses engaged in HSB as a result of being strengthened by comfort interventions, they are more satisfied with health care and demonstrate better health-related and institutional outcome. Employee well-being is defined by the World Health Organization as “the condition of each employee in which they recognize their potential, manage with life pressures, work successfully, and contribute to their community.” Workplace well-being has been identified as a key component in influencing organizational performance. The notion of employee well-being at work promotes the benefits of having a healthy workforce to enterprises.^[23,24]^ Employee well-being at work, according to Athota et al,^[25]^ is the physical and mental health of the work force; consequently, employees should work in a stress-free and physically safe setting. So that, the aim of this study is to determine the extent to which nurses are aware of their comfort, HSB, and their psychological well-being in order to assist staff in avoiding career burnout.

## 2. Methods

### 2.1. Aim of the study

A study to examine out nurses’ awareness of self-comfort and well-being in LTC settings in the state of Kuwait to avoid profession burnout.

#### 2.1.1. Research design.

A descriptive cross-sectional study design was utilized.

### 2.2. Objectives

1. Assess level of nurses’ awareness about self-comfort in LTC settings.
2. Assess level of nurses’ awareness about well-being in LTC settings.
3. Investigate the relationship between nurses’ self-comfort and well-being in LTC settings.

### 2.3. Hypothesis

H1: Nurses in LTC settings will have high awareness of their self-comfort.

H2: Nurses in LTC settings will have high awareness of their well-being.

H3: There is a significant relationship between nurses’ self-comfort and well-being in LTC settings.

### 2.4. Research questions

What is the level of nurses’ perception regarding their comfort and psychological well-being?

Is there a relationship between nurses’ comfort and well-being among LTC nurses?

### 2.5. Setting

This is a cross-sectional multicenter, correlational, descriptive study conducted at LTC facilities in 2 different organizations in Kuwait.

The “Social Welfare Homes Institution” consists of 5 homes and, “The Handicapped Care and Rehabilitation Center.”

- Both institutions were affiliated to Ministry of Social Affairs since they stablished, and in September 27, 2017. The Home for Handicapped Care, the Home for Social Habilitation Care, and Medical Rehabilitation Center transferred to the Public Authority for Disability Affairs, the 3 facilities are one of the buildings situated in the Social Welfare Institution. The 3 facilities provide residential care with social and health services including medical, nursing, physical therapy, dental, and supportive services such as pharmacy, radiology, laboratory, and paramedics. Medical Rehabilitation Center is the facility that provides the healthcare services for the residents.
- The bed capacity for Home for Handicapped care is (276) bed, for the Home for Social Habilitation care is (128) bed, and for the Handicapped care and Rehabilitation Center is (200) bed.
- The Kuwait Center for Mental Health is affiliated to a Ministry of Health, it is the first psychological center in the State of Kuwait, it contains many psychiatric clinics, that is divided into several different specialties: psychological counseling clinics, psychiatric clinics, behavioral disorders clinics, and children’s mental health clinics, the center also provides inpatient services for people with mental problems. The center is a governmental hospital that provides free and supportive services to citizen and expects. The study will include the wards that provide LTC only. This facility has bed capacity of (600) bed.

A sample of 300 nurses was recruited using the following simple random sampling and power analysis technique: the total population is 1350, the confidence level is 95%, and the margin of error is 5%. Problem prevalence is 50%. The maximum sample size is 300. The response rate was 86.7%, with 260 nurses agreeing to fill out the questionnaire. To assess the feasibility of the investigation, 10% of the study sample (n = 30) participated in a pilot study; individuals in the pilot study were eliminated from the study sample. The pilot study revealed that the study instruments were clear and that no changes were required. Data were collected over a 3-month period (from the first of August 2022 to the end of October 2022) by distributing an online questionnaire (in Arabic and English). The questionnaire took around 25 minutes to complete and measured participants’ demographic data, nurses’ knowledge of their comfort, and psychological well-being.

### 2.6. Measurements

Demographic data included age, gender, marital status, qualification, unit, current position, and years of experience. The nurses comfort was assessed using The Nurses Comfort Questionnaire that was developed by Kolcaba,^[26]^ the founder of The Comfort Theory. It consists of 48 items, the items grouped into four dimensions namely; social (15 items, *α* = 0.789), psycho-spiritual (19 items, *α* = 0.749), physical (5 items, *α* = 0.0.791), and environmental (9 items, *α* = 0.789). Researchers computed the average score for the total scale score (*α* = 0.842). Responses were measured on a 6-point Likert scale ranging from 1 = strongly disagree to 6 = strongly agree. The overall score level ranging from 48 to 288; higher scores indicate high level of nurses’ comfort. The levels of comfort were categorized as follows; <50% for low level, from 50% to <75% for moderate level, and ≥75% for high level of comfort. in addition to the Taxonomic Structure of Comfort (comfort Grid) to place the questions on the context in which comfort occur and create a map of questions to indicate the dominant area of comfort as following; Relief—the state of having a specific comfort need met, Ease—the state of calm or contentment, and Transcendence—the state in which one can rise above problems or pain. The researcher fills in the map with own and retained positive and negative items that are specific to the population of the study.

For assessing nurses psychological well-being, researchers used the NIOSH Worker Well-Being Questionnaire (WellBQ) (Chari et al).**^[27]^** This scale consists of 68 items. Researchers computed the average score for the total scale score (*α* = 0.898). The items grouped into 4 sections namely; Work Evaluation and Experience (16 items), Workplace Policies and Culture (14 items), Workplace Physical Environment and Safety Climate (10 items), Health Status (23 items), Home, Community, and Society (5 items), Responses were measured on a 5-point Likert scale ranging from 1 = strongly disagree to 5 = strongly agree. Higher scores indicate high level of well-being. The levels of well-being were arranged as follows; <50% for low level, from 50% to <75% for moderate level, and ≥75% for high level of well-being. Permission was obtained to modify the questionnaire to be suitable for the culture of the study.

### 2.7. Statistical analysis

The mean and standard deviation, as well as numbers and percentages, were used to assess the distributional properties of research variables. Pearson Product-Moment correlations (*r*) were used to evaluate relationships between nurses’ comfort and well-being. The factors in the association research were also investigated using structural equation modeling. The model featured 2 measuring models: 1 for comfort and 1 for well-being, as well as one structural model that related the comfort and well-being of supervisors and nurses. IBM SPSS Statistics version 23 and IBM SPSS AMOS version 23 were used for all statistical analyses. All *P*-values presented are 2-tailed, and for statistical significance, the 0.5 level was chosen.

### 2.8. Ethical considerations

The current study was approved by the Ministry of Health’s Research Ethics Committee, the State of Kuwait’s Public Authority for Disabled Affairs, and Princess Nourah bint Abdulrahman University (IRB: 22-0418) (Kingdom of Saudi Arabia). The administrative authority of the study location gave written permission to conduct the study. The opening page of the questionnaire explained the goal of the study, promised respondents that their responses would be voluntary and confidential, and noted that researchers would consider the completion and submission of the questionnaires as consent to participate.

## 3. Results

After following up with all participants, the response rate was 86.7%. The majority of participants (73.5%) were females and 96.9% of them were married, with more than two-thirds of them being within the age group ranged from 40 to 49. About half of the participants (52.7%) were working at Sabah medical region. As well as, more than half of the participants (57.3%) were working as senior nurse. 62.3% of the participants were working in their job from 10 to 20 years of experience and 52.7% of them were the head of their household. The majority of the participants (83.1%) were from Indian nationality followed by Egyptian nationality (10.8%). More than two thirds of the nursing staff (62.7%) who were working at the medical rehabilitation center, their main workplace was handicapped sections. Sixty-five percent of the study participants had a diploma degree in nursing. Table 1 shows the socio-demographic characteristics of the respondents.

**Table 1:** Socio-demographic characteristics of participants (n = 260).

Socio-demographic characteristics	No	%
Place of work		
South Sabahya	65	25.0
Sabah Medical Region	137	52.7
Kuwait Center for Mental Health	58	22.3
Staff who is working in Medical Rehabilitation Center, please chose your main workplace		
None (for Kuwait Center for Mental Health)	59	22.7
Home for Social Rehabilitation	38	14.6
Handicapped sections	163	62.7
What is your position?		
Assistant Director of Nursing—Senior specialist	3	1.2
Head Nurse—Nurse	5	1.9
Head Nurse—Senior specialist	9	3.5
Nurse—Senior	149	57.3
Nurse—Senior specialist	27	10.4
Nurse	58	22.3
Nurse Assistant	3	1.2
Nursing Specialist	3	1.2
Senior Nursing Technician	3	1.2
How long have you worked in your job?		
<1 yr	2	0.8
1–5 yr	4	1.5
6–10 yr	37	14.2
10–20 yr	162	62.3
More than 20 yr	55	21.2
Age		
20–29	1	0.4
30–39	56	21.5
40–49	160	61.5
50+	43	16.5
Level of education		
Diploma	169	65.0
Nursing certificate after secondary school	10	3.8
Bachelor’s degree	79	30.38
Higher education	2	0.76
Gender		
Male	69	26.5
Female	191	73.5
Nationality		
Egypt	28	10.8
Indian	216	83.1
Kuwait	1	0.4
Philippine	3	1.2
Other	12	4.6
Are you the head of your household?		
No	137	52.7
Yes	123	47.3
What is your current marital status?		
Married	252	96.9
Widowed	7	2.7
Divorced	1	0.4

According to Table 2, the overall nurses’ comfort level was moderate with a mean percent score of 67.55 ± 12.50. The highest percentage of nurses had high Psycho-spiritual comfort (65.4%) with a mean percent score of 77.59 ± 16.08, as the highest mean percent score was related to transcendence (|78.40 ± 18.07). About half of the nurses (52.7%) had moderate level of socio-cultural political comfort with a mean percent score of 68.37 ± 14.90, as the highest mean percent score was related to ease (73.83 ± 16.05). In relation to physical comfort, about half (55.8%) had moderate level of physical comfort with a mean percent score of terms of 67.03 ± 15.21. More than two thirds (71.2%) of the study participants had moderate level of environmental comfort with a mean percent score of 57.23 ± 12.18.

**Table 2:** Mean percent score and level of nurses’ comfort (n = 260).

Nurses’ comfort	% score	Low (<50%)		Moderate (50%–75%)		High (≥75%)	
	Mean ± SD	No	%	No	%	No	%
Physical comfort	67.03 ± 15.21	26	10.0	145	55.8	89	34.2
Relief	65.48 ± 16.77	37	14.2	126	48.5	97	37.3
Ease	65.13 ± 19.0	34	13.1	142	54.6	84	32.3
Transcendence	70.48 ± 21.70	37	14.2	94	36.2	129	49.6
Psycho-spiritual comfort	77.59 ± 16.08	16	6.2	74	28.5	170	65.4
Relief	76.99 ± 20.13	21	8.1	70	26.9	169	65.0
Ease	77.35 ± 18.60	22	8.5	60	23.1	178	68.5
Transcendence	78.40 ± 18.07	14	5.4	66	25.4	180	69.2
Environmental comfort	57.23 ± 12.18	54	20.8	185	71.2	21	8.1
Relief	52.96 ± 20.11	104	40.0	104	40.0	52	20.0
Ease	52.40 ± 16.14	97	37.3	139	53.5	24	9.2
Transcendence	66.33 ± 18.33	35	13.5	128	49.2	97	37.3
Socio-cultural political comfort	68.37 ± 14.90	23	8.8	137	52.7	100	38.5
Relief	66.48 ± 18.50	41	15.8	111	42.7	108	41.5
Ease	73.83 ± 16.05	20	7.7	80	30.8	160	61.5
Transcendence	64.79 ± 21.46	50	19.2	114	43.8	96	36.9
Overall, nurses’ comfort	67.55 ± 12.50	18	6.9	163	62.7	79	30.4

SD = standard deviation.

In relation to Table 3, the overall nurses’ well-being level was low with a mean percent score of 49.58 ± 6.93. The highest percentage of nurses had moderate level of work evaluation and experience domain of well-being (76.9%) with a mean percent score of 58.26 ± 9.91, as the highest mean percent score was related to work-related fatigue and engagement (69.65 ± 13.18), followed by work-related positive affect (69.05 ± 27.65). About two thirds of nurses (62.7%) had low level of Workplace Policies and Culture domain of well-being with a mean percent score of 44.88 ± 11.73, as the highest mean percent score was related to supportive work culture and trust (72.69 ± 22.26), followed by health culture at work (72.69 ± 22.26). In relation to workplace physical environment and safety climate, more than half of nurses (57.3%) had low level of it with a mean percent score of 47.37 ± 14.73; as the highest mean percent score was related to physical work environment satisfaction (64.71 ± 24.48) and followed by workplace safety climate (62.37 ± 26.07). Hundred percent of nurses perceived that they had low level of health status domain of well-being with a mean percent score of 14.91 ± 7.71. Furthermore, about half of nurses (50.4%) had low level of well-being in relation to home, community, and society domain with a mean percent score of 48.45 ± 18.58; the highest mean percent score was related to life satisfaction, financial insecurity, and support outside of work with a mean percent score of 62.28 ± 13.88.

**Table 3:** Mean percent score and level of nurses’ well-being (n = 260).

Well-being	Mean % score	Low (<50%)		Moderate (50%–75%)		High (≥75%)	
	Mean ± SD	No	%	No	%	No	%
Work evaluation and experience	58.26 ± 9.91	52	20.0	200	76.9	8	3.1
Work satisfaction, support, security, autonomy, and workload	68.36 ± 15.55	30	11.5	132	50.8	98	37.7
Work-related positive affect	69.05 ± 27.65	66	25.4	39	15.0	155	59.6
Work-related negative affect	22.71 ± 20.33	226	86.9	27	10.4	7	2.7
Work-related fatigue and engagement	69.65 ± 13.18	18	6.9	111	42.7	131	50.4
Workplace policies and culture	44.88 ± 11.73	163	62.7	97	37.3	0	0.0
Supportive work culture and trust	72.69 ± 22.26	31	11.9	108	41.5	121	46.5
Health culture at work	66.93 ± 29.85	52	20.3	90	35.2	114	44.5
Availability of job benefits	51.66 ± 21.78	116	44.8	102	39.4	41	15.8
Availability of health programs at work	28.81 ± 26.34	192	75.3	43	16.9	20	7.8
Work to non-work conflict	25.29 ± 21.25	205	78.8	48	18.5	7	2.7
Workplace schedule flexibility	56.54 ± 21.27	60	23.1	153	58.8	47	18.1
Workplace physical environment and safety climate	47.37 ± 14.73	149	57.3	109	41.9	2	0.8
Workplace safety climate	62.37 ± 26.07	71	27.3	105	40.4	84	32.3
Physical work environment satisfaction	64.71 ± 24.48	53	20.4	116	44.6	91	35.0
Discrimination	11.84 ± 19.53	241	92.7	14	5.4	5	1.9
Work-related physical violence and, bullying	8.59 ± 21.84	242	93.1	11	4.2	7	2.7
Health status	14.91 ± 7.71	260	100.0	0	0.0	0	0.0
Overall health	75.23 ± 17.36	16	6.2	85	32.7	159	61.2
Chronic health conditions	7.18 ± 10.22	258	99.2	2	0.8	0	0.0
Insomnia	4.04 ± 16.23	243	93.5	13	5.0	4	1.5
Overall stress	26.09 ± 21.60	210	80.8	40	15.4	10	3.8
Poor mental health	10.67 ± 15.90	250	96.2	9	3.5	1	0.4
Tobacco use	0.92 ± 4.85	260	100.0	0	0.0	0	0.0
Physical activity	50.29 ± 22.13	89	34.2	90	34.6	81	31.2
Healthy diet	48.85 ± 17.72	99	38.1	144	55.4	17	6.5
Sleep hours	38.85 ± 17.45	145	55.8	88	33.8	27	10.4
Sleep at work	29.54 ± 14.75	232	89.2	23	8.8	5	1.9
Cognitive functioning and work limitations	10.80 ± 17.21	231	93.5	16	6.5	0	0.0
Productivity	7.88 ± 15.34	251	96.5	5	1.9	4	1.5
Work-related injury and injury consequence	6.92 ± 23.04	236	90.8	12	4.6	12	4.6
Home, community, and society	48.45 ± 18.58	131	50.4	111	42.7	18	6.9
Life satisfaction, financial insecurity, and support outside of work	62.28 ± 13.88	24	9.2	169	65.0	67	25.8
Activities outside of work	46.25 ± 22.82	139	53.7	96	37.1	24	9.3
Overall well-being	49.58 ± 6.93	124	47.7	136	52.3	0	0.0

SD = standard deviation.

Regarding the correlation matrix in Table 4, a strong, positive, and significant correlation were noticed overall nurses’ comfort and its domains with the overall nurses’ well-being and its domain (*P* < 0.001).

**Table 4:** Correlation matrix between nurses’ comfort and well-being (n = 260).

	Nurses’ comfort					Well-being					
	Physical	Psycho-spiritual	Environmental	Socio-cultural political	Overall, nurses’ comfort	Work evaluation and experience	Workplace policies and culture	Workplace physical environment and safety climate	Health status	Home, community, and society	Overall well-being
Physical											
*r*											
*P*											
Psycho-spiritual											
*r*	0.682*										
*P*	<.001*										
Environmental											
*r*	0.594*	0.498*									
*P*	<.001*	<.001*									
Socio-cultural political											
*r*	0.708*	0.748*	0.575*								
*P*	<.001*	<.001*	<.001*								
Overall, nurses’ comfort											
*r*	0.880*	0.874*	0.756*	0.895*							
*P*	<.001*	<.001*	<.001*	<.001*							
Work evaluation and experience											
*r*	0.291*	0.375*	0.331*	0.368*	0.400*						
*P*	<.001*	<.001*	<.001*	<.001*	<.001*						
Workplace policies and culture											
*r*	0.484*	0.554*	0.470*	0.628*	0.628*	0.380*					
*P*	<.001*	<.001*	<.001*	<.001*	<.001*	<.001*					
Workplace physical environment and safety climate											
*r*	0.399*	0.341*	0.351*	0.437*	0.447*	0.354*	0.469*				
*P*	<.001*	<.001*	<.001*	<.001*	<.001*	<.001*	<.001*				
Health status											
*r*	−0.439*	−0.510*	−0.312*	−0.479*	−0.517*	−0.179*	−0.319*	−0.171*			
*P*	<.001*	<.001*	<.001*	<.001*	<.001*	.004*	<.001*	.006			
Home, community, and society											
*r*	−0.022	0.045	−0.027	−0.002	0.001	0.252*	0.078	0.142*	0.052		
*P*	.721	.467	.665	.978	.991	<.001*	.207	.022*	.402		
Overall well-being											
*r*	0.209*	0.255*	0.259*	0.293*	0.296*	0.706*	0.530*	0.573*	0.198*	0.632*	
*P*	.001*	<.001*	<.001*	<.001*	<.001*	<.001*	<.001*	<.001*	.001	<.001*	

*r* = Pearson coefficient.

* Statistically significant at *P* ≤ 0.05.

Table 5 shows that there was a positive significant correlation between nurses’ comfort and their level of education (*F* = 3.550 and *P* = .015). As well as, there was no significant correlation between nurses’ comfort and their age and gender. Furthermore, Table 6 shows that there was no significant correlation between nurses’ well-being and their age, level of education, and gender. However, there was positive significant correlation between work evaluation an experience domain of well-being and nurses’ level of education (*H* = 9.474, *P* = .024). In addition, there was a significant positive correlation between workplace physical environment and safety climate and nurses’ age (*H* = 7.029, *P* = .030). As well as, there was a significant correlation between health status domain of well-being and nurses’ age, level of education and gender (*H* = 8.210, *P* = .016, *H* = 10.382, *P* = .016, *U* = 4750, *P* = .001), respectively. Furthermore, there was a significant positive correlation between home, community, and society domain of well-being and nurses’ level of education (*H* = 10.028, *P* = .018).

**Table 5:** Relationship between the nurses’ comfort and their demographic data.

	Nurses’ comfort (% score)				
	Physical	Psycho-spiritual	Environmental	Socio-cultural political	Overall, nurses’ comfort
	Mean ± SD	Mean ± SD	Mean ± SD	Mean ± SD	Mean ± SD
Age					
20–39	64.62 ± 17.20	73.54 ± 18.13	56.99 ± 11.43	66.52 ± 16.42	65.42 ± 13.77
40–49	67.17 ± 15.34	78.08 ± 15.88	56.34 ± 12.60	68.20 ± 14.89	67.45 ± 12.64
50+	69.73 ± 11.25	81.12 ± 12.89	60.85 ± 11.09	71.43 ± 12.51	70.78 ± 9.37
*F*(*P*)	1.402 (.248)	2.961 (.054)	2.362 (.096)	1.362 (.258)	2.300 (.102)
Level of education					
Diploma	68.56 ± 13.00	79.93 ± 12.59	58.30 ± 10.81	70.36 ± 12.92	69.29 ± 9.82
Nursing certificate after secondary school	68.17 ± 10.50	76.08 ± 16.61	55.50 ± 7.94	69.33 ± 10.07	67.27 ± 9.61
Bachelor’s degree	64.24 ± 18.87	72.86 ± 20.69	55.37 ± 13.92	64.24 ± 17.66	64.18 ± 16.00
Higher education	53.33 ± 23.96	73.33 ± 29.22	52.08 ± 30.98	61.25 ± 29.92	60.00 ± 27.91
*F*(*P*)	2.577 (.054)	3.645* (.013*)	1.349 (.259)	3.376* (.019*)	3.550* (.015*)
Gender					
Male	66.55 ± 15.12	73.89 ± 16.78	59.52 ± 10.64	66.18 ± 13.89	66.53 ± 11.97
Female	67.21 ± 15.28	78.92 ± 15.65	56.40 ± 12.61	69.15 ± 15.21	67.92 ± 12.69
*t*(*P*)	0.309 (.757)	2.246* (.026*)	1.828 (.069)	1.421 (.156)	0.791 (.430)

ANOVA = Analysis of variance, *F* = *F* for 1 way ANOVA test, *P* = *P* value for comparison between the studied categories, SD = standard deviation, *t* = Student *t* test.

* Statistically significant at *P* ≤ .05.

**Table 6:** Relation between nurses’ well-being and their demographic data.

	Well-being (% score)											
	Work evaluation and experience		Workplace policies and culture		Workplace physical environment and safety climate		Health status		Home, community, and society		Overall, well-being	
	Mean ± SD	Median	Mean ± SD	Median	Mean ± SD	Median	Mean ± SD	Median	Mean ± SD	Median	Mean ± SD	Median
Age												
20–39	57.54 ± 9.44	56.19	44.45 ± 12.93	43.08	48.75 ± 15.20	45.24	17.23 ± 7.82	16.39	51.72 ± 19.38	51.85	50.82 ± 7.69	51.20
40–49	58.53 ± 9.92	59.52	44.46 ± 11.79	44.62	45.65 ± 13.88	45.24	14.54 ± 7.85	12.30	48.63 ± 17.24	48.15	49.26 ± 6.43	50.00
50+	58.23 ± 10.60	59.05	47.01 ± 9.70	49.23	51.94 ± 16.28	52.38	13.21 ± 6.45	11.48	43.45 ± 21.53	44.44	49.13 ± 7.61	49.40
*H*(*P*)	0.445 (.801)		1.360 (.507)		7.029* (.030*)		8.210* (.016*)		5.227 (.073)		2.609 (.271)	
Level of education												
Diploma	59.46 ± 10.01	60.00	46.12 ± 11.12	47.69	47.86 ± 14.80	47.62	13.97 ± 7.04	11.48	49.78 ± 18.38	51.85	50.13 ± 6.83	50.90
Nursing certificate after secondary school	54.67 ± 10.26	59.05	44.92 ± 13.19	46.15	43.10 ± 13.95	46.43	12.30 ± 7.40	11.07	31.48 ± 14.63	32.41	44.22 ± 7.17	47.01
Bachelor’s degree	56.44 ± 9.48	57.14	42.52 ± 12.50	43.08	46.63 ± 14.66	45.24	17.23 ± 8.65	15.57	47.52 ± 18.76	46.30	49.15 ± 7.02	49.40
Higher education	51.67 ± 1.63	51.90	38.08 ± 14.01	39.23	51.79 ± 17.64	50.00	16.60 ± 9.61	15.16	52.78 ± 15.01	56.48	48.05 ± 2.14	48.05
*H (P*)	9.474* (.024*)		4.987 (.173)		1.219 (.748)		10.382* (.016*)		10.028* (.018*)		7.634 (.054)	
Gender												
Male	56.59 ± 10.29	59.05	43.57 ± 11.69	43.08	47.58 ± 15.52	45.24	17.51 ± 7.91	16.39	50.38 ± 22.08	50.00	50.09 ± 7.48	50.60
Female	58.87 ± 9.72	59.05	45.36 ± 11.75	46.15	47.29 ± 14.47	47.62	13.97 ± 7.44	11.48	47.76 ± 17.16	48.15	49.40 ± 6.73	50.00
*U*(*P*)	5911.50 (.205)		5892.00 (.192)		6534.0 (.917)		4750.0* (.001*)		6027.0 (.293)		6096.0 (.357)	

*H* = *H* for Kruskal Wallis test, *P* = *P* value for comparison between the studied categories, SD = standard deviation, *U* = Mann Whitney test.

* Statistically significant at *P* ≤ .05.

To validate the relation between nurses’ comfort and their well-being, a regression analysis was performed, with nurses’ comfort serving as the independent variable and well-being serving as the dependent variable (Table 7). The regression study revealed that nurses’ perception of their comfort may predict their well-being (*P* < .001) and explained 34% of the variation in nurses’ well-being (*R*^2^ = 0.470, *β* = 0.428, *F* = 34.762, *P* < .001).

**Table 7:** Hierarchical linear regression analysis (stepwise) showing the effect of nurses’ comfort on their well-being.

Variable	*B*	*β*	*t*	*P*	95% CI	
					LL	UL
Wellbeing	0.349	0.428	5.265*	<0.001*	−0.481	−0.218
*R*^2^ = 0.470, *F* = 34.762*, *P* < .001*						

*B* = unstandardized coefficients, *F, P* = *F* and *P* values for the model, LL = lower limit, *R*^2^ = coefficient of determination, *t* = *t* test of significance, UL = upper limit, *β* = standardized coefficients.

* Statistically significant at *P* ≤ .05.

## 4. Discussion

Nursing has always been about providing individuals with the most comfort and convenience possible via care. Shares of comfort-oriented care provided by nurses to citizens generate holistic responses to complex stimuli, leading to improved comfort while seeking behavior directed at strengthening health.^[28,29]^ This care is provided through interpersonal processes inherent in self-construction as well as the situations faced both naturally and socially from birth to death.^[30]^ Numerous nursing researchers have explored comfort care as a phenomenon.^[31]^ However, in many proposals, the concepts of comfort, convenience, and welfare are used as synonyms when describing the action of caring as thought, as caudation made as a result of the act of caring.^[32]^

The current study looked at the connection between nurses’ knowledge of self-comfort and well-being in LTC settings in Kuwait. The findings confirmed that nurses’ comfort was considerably positively associated to their well-being, with comfort accounting for 34% of the variance in nurses’ well-being. The topic of comfort among nurses has received little attention because most studies have concentrated on the nursing practice of providing comfort to their patients rather than analyzing their degree of comfort or their knowledge of their comfort level and how it affects their well-being and associated domains. The present research is one of the first to look at patient comfort and well-being in the nursing industry, specifically in Kuwait. The findings may help nursing leaders manage the environmental characteristics, physical resources, socio-cultural environment, and psycho-spiritual comfort that support nursing well-being through work satisfaction, security, safety work climate, and autonomy, which may improve nurses’ work engagement, health status, life and job satisfaction, and job security.

The current study found that the nurses surveyed experienced a modest level of self-comfort. This is consistent with previous studies on nurses’ comfort, which found that nursing staff reported moderate levels of comfort, which may be due to their workload of documentation and work-related practices, as well as a staff shortage that was exacerbated by the COVID-19 pandemic.^[33–35]^ Nurses are leaving the field because they believe their salaries are insufficient for the duties they perform, and the administration has no plans to hire more employees. Furthermore, the inflexibility of the work regulation had made nurses concerned about the consequences of making a mistake at work because the residents were deemed a vulnerable population that required special care.

The psycho-spiritual climate in the healthcare study setting may have contributed to the nurses’ moderate degree of comfort, since the nurses may feel competent and confident, but they may also feel they don’t belong at work, and they may not feel healthy at work. Furthermore, socio-cultural aspects such as feeling calm, receiving adequate support from their nurse managers, and being driven to perform their best can all contribute to their moderate level of comfort. That is, nurses are encouraged to strive for excellence and to engage in ongoing professional development activities. These tactics appear to be beneficial in assisting nurses in developing their talents, strengthening their dedication to their employment, and maximizing their maximum ability with effective and efficient use of existing resources.

Furthermore, the study results suggested that the nurses surveyed had a poor degree of happiness. This aligns with prior research on nurses’ well-being.^[36,37]^ They discovered that nurses had a low level of mental and psychological well-being as a result of higher patient admission rates and associated workload, as well as a stressful work environment. Nurses may have low sleep hours, low physical activities due to long working hours shifts, limited cognitive functioning at work, the incidence of work-related injuries and their consequences, the overall stress they face in different situations in nursing practice, and insomnia due to limited sleep hours. Because some of the buildings were outdated and needed to be replaced, the unsafe work environment played a significant part in the staff’s low level of well-being. Again, workplace regulations and culture, combined with a lack of access to various health programs at work, can lead to a poor degree of well-being.

Previous research found that employees with a high level of comfort can change their work environment proactively to align job policies, culture, and resources with their abilities, health, and needs, which indirectly improves their satisfaction, commitment, motivation, and work engagement.^[38,39]^ Future multicenter and comparative research is needed to validate the influence of workplace culture and work climate on nurses’ comfort and well-being.

This study corroborated prior research on the association between nurses’ comfort and well-being,^[40]^ which found that higher well-being was associated with higher comfort level. This may be due to the effect of psycho-social work climate on the nurse’s health and well-being, as when they feel supported and guided by their managers, and feel the value of their work, they will be encouraged and motivated to manage their stressful working environment by participating in decision making for arranging working hours to reduce the workload and manage it properly to make the work more productive and motivating, positive work climate.

### 4.1. Implications for nursing management

Nurse managers should maintain a variety of behaviors that support a comfortable and healthy work environment. First, nurse managers should examine and change the work policy to reflect the conditions of nurses, particularly in light of the resignations and decrease in the number of nurses following the Covid-19 outbreak. While also fostering a healthy work atmosphere that allows nurses to give the greatest levels of compassionate care while also feeling fulfilled at work. In addition, nurse managers should conduct wellness and resilience training programs to lower the number of employees who feel overburdened at work on a regular basis. Furthermore, a review of nurse staffing for recruitment is required. Finally, nurse managers can undertake a free assessment of the institution in order to identify the evidence-based resources required to create and maintain a healthy work environment.

### 4.2. Strengths and limitations of the study

This study’s findings considerably added to existing studies on nurses’ comfort and welfare in the Covid-19 era. The study, however, should be viewed in light of its limitations. Because the individuals were recruited from a specific location for convenience, the results’ generalizability is limited. Furthermore, the current findings were prone to response bias and subjectivity because they were based on self-reported data. Furthermore, this study merely found correlations between study variables; no causal relationship was found. Longitudinal, experimental, and multi-site research may assist to resolve these constraints in the future. The current study had various advantages, including the ability to test multiple factors simultaneously in a population sample, resulting in more trustworthy data that was less subject to the possible biases of case series and case reports. A longer inquiry could have benefited from a longer follow-up. Finally, no claims were made about the link between the variables in the study; the study’s purpose was to investigate the relationship between variables. Future study should concentrate on specific tactics or therapies for dealing with the comfort and well-being of nurses. Future study can also assess nurses’ compassion fatigue, organizational commitment, and the work environment’s readiness to preserve nurses’ wellness and comfort.

## 5. Conclusions

The current study looked at the connection between nurses’ knowledge of self-comfort and well-being in LTC settings in Kuwait. The findings showed that nurses’ perceptions of their comfort might predict their happiness and accounted 34% of the difference in nurses’ happiness. The association between nurses’ comfort and well-being is strong across all areas of comfort and well-being. Future study is needed to confirm the comfort and well-being scale’s validity among Saudi nurses. Future research is required to uncover techniques that effectively improve nurses’ comfort and various images of well-being in the workplace.

## Acknowledgments

The authors acknowledge the participants for accepting to participate in the study.

## Author contributions

**Conceptualization:** Hasna’a Salem Saed Boswihi, Sally Farghaly Abdelaliem.

**Formal analysis:** Hasna’a Salem Saed Boswihi, Sally Farghaly Abdelaliem.

**Methodology:** Hasna’a Salem Saed Boswihi, Sally Farghaly Abdelaliem.

**Project administration:** Hasna’a Salem Saed Boswihi, Sally Farghaly Abdelaliem.

**Investigation:** Sally Farghaly Abdelaliem.

**Supervision:** Sally Farghaly Abdelaliem.

**Validation:** Sally Farghaly Abdelaliem.

**Writing—original draft:** Sally Farghaly Abdelaliem.

**Writing—review & editing:** Sally Farghaly Abdelaliem.
